# Differential effects of changes in cardiorespiratory fitness on worst- and best- school subjects

**DOI:** 10.1038/s41539-021-00086-8

**Published:** 2021-04-01

**Authors:** Toru Ishihara, Noriteru Morita, Toshihiro Nakajima, Koji Yamatsu, Koichi Okita, Masato Sagawa, Keita Kamijo

**Affiliations:** 1 Graduate School of Human Development and Environment, Kobe University, Kobe, Hyogo Japan; 2 Department of Sports Cultural Studies, Hokkaido University of Education, Iwamizawa, Hokkaido Japan; 3 Department of Teachers Training, Hokkaido University of Education, Sapporo, Hokkaido Japan; 4 Faculty of Education, Saga University, Saga, Saga Japan; 5 Department of Sport Education, Hokusho University, Ebetsu, Hokkaido Japan; 6 University of Tsukuba, Tsukuba, Ibaraki Japan

**Keywords:** Human behaviour, Education

## Abstract

Accumulating evidence shows a beneficial association between physical fitness and school children’s academic performance. However, several other studies have failed to demonstrate such an association. We reanalyzed data of a two-year longitudinal study of the association between changes in cardiorespiratory fitness and academic performance of school children by focusing on intra-individual variability in grade points as a possible source of this discrepancy. We analyzed data from 469 junior high school students to examine if improvements in cardiorespiratory fitness had a differential effect on an individual student’s worst and best grade points. Results indicated that improvements in physical fitness were associated with an improvement in the worst grade points. On the contrary, we did not observe a similar longitudinal association with the best grade points. These findings suggest that improving cardiorespiratory fitness improves the worst grade points of an individual, selectively. We suggest that intra-individual variability in grade points might moderate the association between physical fitness and academic performance changes.

## Introduction

The epidemic of physical inactivity and the corresponding reduction in physical fitness during childhood has become a global public health problem^1,2^. A large body of cross-sectional studies has consistently found a positive association between physical fitness, including several of its dimensions, such as cardiorespiratory fitness, muscular fitness, and motor fitness, and the academic performance of school children^3–6^, measured by academic subjects’ grade points (GP), or standardized academic test scores. These findings indicate the crucial role of regular physical activity and the associated improvements in physical fitness on the healthy development of the brain. Moreover, several longitudinal studies, including observational studies, randomized controlled trials (RCTs), and cluster RCTs have supported these cross-sectional findings by also demonstrating a positive causal link between improvements in physical fitness associated with regular physical activity and academic performance^7–11^. However, several recent large-scale cluster RCTs have reported no effect of physical activity interventions on academic performance^12–14^, casting doubt on the causality in this relationship. Multiple factors might cause the discrepant findings of longitudinal studies, including participant characteristics (e.g., sex) and the characteristics of the physical activity investigated (e.g., type and duration)^15^. We focused on intra-individual variability in GP for academic subjects, among other factors, to identify possible moderators of the association between physical fitness and academic performance.

Most cross-sectional studies have demonstrated a positive association between childhood fitness and academic performance. Nevertheless, this association might differ based on the subject. For example, several studies have reported a positive association between cardiorespiratory fitness and mathematics, but not with reading^16,17^, suggesting that only certain subjects might benefit from the effects of physical fitness. Another study, however, has reported the opposite association, indicating that cardiorespiratory fitness is significantly associated with reading, but not with mathematics^18^. Similarly, longitudinal studies have shown contradictory findings by indicating different changes for different academic subjects^9,19^. We assumed that this discrepancy might be partly due to the baseline performance of a student. Ishihara, Drollette, Ludyga, Hillman, and Kamijo^20^ reanalyzed three RCTs to examine whether the effects of physical activity interventions on executive function, which refers to prefrontal dependent goal-directed cognitive processes^21^, are moderated by the baseline performance. A large body of evidence has shown that executive function predicts both GP and standardized academic test scores^22^, indicating an association between physical fitness and academic performance. Ishihara et al.^20^ indicated that improvements in executive function resulting from physical activity interventions were greater for individuals with lower baseline cognitive performance. Likewise, subgroup analysis of a large-scale cluster RCT^13^ that found no effects of physical activity interventions on academic performance, however, also indicated that children with the lowest tertile group for math performance at baseline improved their academic performance following the intervention, which was not the case for children in the higher tertile groups. Therefore, a low baseline performance might be associated with greater benefits from physical activity interventions. These findings suggest that inter-individual differences in baseline performance might moderate the effects of physical activity interventions on academic performance.

From the alternative perspective of baseline performance, intra-individual variability in GPs might potentially affect the longitudinal association between improved physical fitness and academic performance. Studies focusing on inter-individual differences in baseline performance^13,20^ suggest that an individual’s worst school subjects are more sensitive to the beneficial effects of improved physical fitness than the best subjects. Moreover, the two studies cited above also indicated that physical activity interventions did not cause a deterioration of executive or academic performance even in individuals with high baseline performance, suggesting that increasing the time spent on physical activities does not detract from academic performance, which was also corroborated in a systematic review by Donnelly et al.^23^. Therefore, findings at the intra-individual level suggest that improving physical fitness does not reduce the best grades of an individual student.

Recent studies in this field have employed rigorous designs controlling for potential confounders such as socioeconomic status (SES), which might be a possible source of inconsistencies in early studies^23^. Nevertheless, most studies have failed to consider out-of-school learning activities, which is a critical factor affecting academic performance. Out-of-school learning activities, such as doing homework, attending cram schools, and private tutoring, are known to influence academic performance directly^3,24^. Therefore, it is essential to examine whether the expected association between physical fitness and academic performance would be observed after controlling for out-of-school learning time.

The present study reanalyzed data from a two-year longitudinal study^11^ to investigate the association between changes in physical fitness and academic performance of junior high school children by focusing on intra-individual variability in school grades. We specifically examined if improvements in cardiorespiratory fitness had differential effects on an individual’s worst and best GPs. We focused on cardiorespiratory fitness, which several studies have indicated was positively associated with academic performance^3–5,7,10,11^, although the exact changes in fitness components that most contribute to improving academic performance remain unclear. Moreover, we rigorously controlled for potentially significant confounding variables, including out-of-school learning time and SES, when examining the association between cardiorespiratory fitness and academic performance. We predicted that physical fitness improvements would be associated with an improvement in the lowest GP, whereas it would have weak or no association with the highest GP.

## Results

**Fig. 1:**
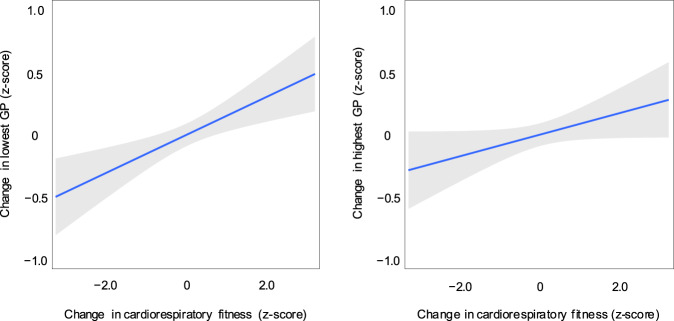
The associations between changes in cardiorespiratory fitness and changes in the lowest (left panel) and highest (right panel) GPs. Regression lines are shown with 95% confidence bands (shaded areas).

The results of Pearson’s correlation analyses are presented in Table 1. It can be seen that changes in cardiorespiratory fitness were not associated with changes in learning time. However, changes in learning time were positively associated with changes in the lowest and highest GPs, whereas changes in cardiorespiratory fitness were positively associated with only changes in the lowest GP. No association was found between changes in cardiorespiratory fitness and the highest GP. The associations between changes in cardiorespiratory fitness and changes in the lowest and highest GPs are illustrated in Fig. 1. Multiple regression analyses indicated that these associations between changes in cardiorespiratory fitness with changes in the lowest and highest GPs remained unchanged (Cohen’s *f*^2^ = 0.02, 1−β = 0.87 and Cohen’s *f*^2^ = 0.003, 1−β = 0.22, respectively) after controlling for the household income, maternal education, sex, change in BMI, and change in learning time (Table 2).

**Table 1:** Results of Pearson’s correlation analyses.

	1	2	3	4	5	6	7
1. Change in cardiorespiratory fitness	–						
2. Change in lowest GP	0.15*	–					
3. Change in highest GP	0.09	0.35*	–				
4. Household income	0.12*	0.01	0.04	–			
5. Maternal education	0.12*	0.09	−0.06	0.21*	–		
6. Sex	0.35*	0.04	0.07	0.08	0.13*	–	
7. Change in BMI	−0.27*	0.04	−0.04	−0.01	−0.04	−0.26*	–
8. Change in learning time	0.05	0.17*	0.21*	0.09	0.04	−0.10*	0.05

*GP* grade point, *BMI* body mass index.

*p < 0.05.

**Table 2:** Results of multiple regression analyses predicting changes in the lowest and highest grade points.

Variables	Change in lowest GP				Change in highest GP			
	β	SE	95% CI	*p*	β	SE	95% CI	*p*
Change in cardiorespiratory fitness	0.15	0.05	0.06 to 0.25	0.002	0.06	0.05	−0.04 to 0.16	0.25
	Adjusted Δ*R*^2^ = 0.02				Adjusted Δ*R*^2^ = 0.003			
Confounders								
Household income	−0.04	0.05	−0.14 to 0.07	0.48	0.01	0.05	−0.09 to 0.12	0.81
Maternal education	0.07	0.05	−0.03 to 0.17	0.17	−0.10	0.05	−0.20 to 0.008	0.07
Sex	0.01	0.05	−0.08 to 0.11	0.78	0.08	0.05	−0.02 to 0.18	0.10
Change in BMI	0.06	0.06	−0.05 to 0.18	0.26	−0.009	0.06	−0.12 to 0.10	0.88
Change in learning time	0.16	0.05	0.07 to 0.25	<0.001	0.22	0.05	0.13 to 0.31	<0.001

*BMI* body mass index, *GP* grade point, *SE* standard error.

## Discussion

This study’s main findings corroborated the study’s hypothesis by indicating that improvements in physical fitness were associated with an improvement in the worst GPs, whereas a similar longitudinal association was not observed for the highest GP. These findings are consistent with the results of RCTs that focused on inter-individual differences in baseline performance and reported that lower baseline performance is associated with greater benefits from physical activity interventions^13,20^. The present study extended this line of research by showing that the longitudinal association between improvements in physical fitness and academic performance is moderated by intra-individual variability in academic grades. Stated differently, these findings suggest that the beneficial association between physical fitness and the lowest GP could be observed even in students with high baseline performance.

Moreover, this study suggests that the null effect of physical activity interventions on academic performance observed in previous RCTs^12–14^ could be partially explained by the difficulty of academic tests. The beneficial effects of physical activity interventions on academic performance might not be detectable when the overall test scores of an individual are relatively high. Also, this study indicated that physical fitness changes were not statistically related to the highest GP, which supports the position of the American College of Sports Medicine that increasing physical activities does not negatively influence academic performance^23^.

The present study provides novel findings regarding out-of-school learning time. Not surprisingly, increased out-of-school learning time increased both the lowest and highest GPs. More importantly, the longitudinal association between changes in cardiorespiratory fitness and the lowest GP was independent of out-of-school learning time, suggesting that the observed increase in the lowest GP might have resulted from changes in classroom behaviors. Several RCTs have shown that physical activity interventions leading to increased cardiorespiratory fitness improve prefrontal cortex dependent executive function^25–28^, demonstrating a bidirectional relationship with academic performance^29^. Furthermore, executive function is likely to have critical functions in sustained attention^30^ and academic motivation^31^. Although speculative, improvements in fitness might improve students’ classroom behaviors, including the ability to concentrate on learning, and the motivation for learning might collectively increase the lowest GP. In contrast, no association was observed in the highest GP, probably because each participant might have been more motivated for the best subject even at baseline. Several studies have examined the effects of physical activity on on-task classroom behaviors. These studies suggest that on-task classroom behaviors can be improved immediately after a single bout of physical activity (i.e., acute positive effects)^32^. However, Szabo-Reed et al.^33^ also reported that a 3-year physical activity intervention did not change on-task classroom behaviors (i.e., no long-term effects). To date, studies on the long-term effects of physical activity on classroom behaviors are scarce, and more research evaluating the effects of physical activity on classroom behaviors is required in the future to clarify how fitness improvements selectively influence an individual’s worst academic subjects.

Specific limitations of this study should be noted. Firstly, the possible effects of catch-up growth cannot be ruled out because this study used an observational design. From the perspective of inter-individual differences in baseline performance, students with low baseline performance and relatively low physical fitness could experience greater improvements in academic performance and physical fitness at follow-up due to catch-up growth. However, the present study focused on intra-individual variability, which indicated beneficial associations between increased physical fitness and the lowest GP, even in students with a high baseline score. Therefore, we believe that the observed increase in the lowest GP is mainly attributed to improvements in physical fitness rather than catch-up growth. Secondly, this study used cardiorespiratory fitness as a proxy for engagement in regular physical activities. It is unclear whether physical fitness improvements caused by the effects of development, or by physical activity per se, are critical for improving academic performance. Furthermore, investigations assessing both physical fitness and physical activity levels are required to clearly distinguish the effects of regular physical activities from developmental effects. Thirdly, the present study only focused on cardiorespiratory fitness. A recent meta-analysis has suggested that the positive effects of physical activity interventions on executive function, which is pivotal for academic performance^34,35^, are different for different types of physical activity, with coordinative training being more effective than cardiorespiratory and resistance training^15^. It is suggested that future research examine whether the longitudinal relationship between improvements in physical fitness and intra-individual variability of academic subjects differs according to the components of fitness. Fourthly, the GP system varies among countries. The present study only targeted Japanese junior high schools, in which the subject teacher evaluates the GP for each academic subject. The Japanese subject teachers base their assessments on a wide variety of perspectives, including class activities, reflection papers, and paper test scores. Therefore, we believe that the findings of this study can be generalized to other GP assessment systems to a certain extent. Fifthly, none of the participants were receiving special education services for cognitive or attentional disorders. Nevertheless, the lack of assessment and information regarding the participants’ mental health, learning disabilities, or neurological disorders is another limitation of the present study. As a result, we could not determine whether neuropsychological variables cofounded the observed longitudinal associations between changes in fitness and GPs or whether these findings could be generalized to children with psychopathologies. Finally, the possibility of selection bias in the sample cannot be ruled out because approximately one-fourth of students were not included in the study due to missing informed consent.

This two-year longitudinal study demonstrated that a positive association between improvements in physical fitness and academic performance was strongly observed for an individual’s worst GPs. Furthermore, the findings of this study regarding the highest GPs support the new perspective that the increase in physical fitness associated with regular physical activity has no adverse effects on academic performance. In conclusion, this study highlights the importance of childhood fitness for healthy brain development and academic success.

## Method

### Participants

**Fig. 2:**
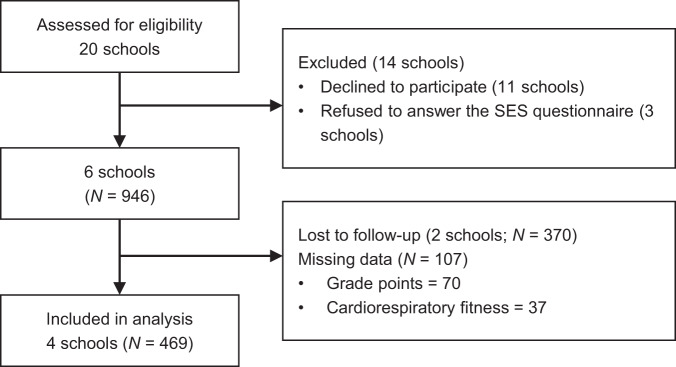
Flow diagram of participant recruitment and follow-up procedures.

Figure 2 shows the flow diagram of the participant recruitment and follow-up processes. We requested 20 public junior high schools in Sapporo, which is the prefectural capital of Hokkaido in northern Japan, and cities near Sapporo, for permission to recruit seventh-grade students (i.e., 12–13 years old). Eleven schools declined to participate, and three schools refused to respond to the SES questionnaire. Two schools were excluded from the analysis due to the lack of follow-up. Only students whose parents or guardians returned the signed informed consent form were included in this study (consent rate = 74%). Finally, the data of participants that completed the two-year study period (*N* = 469, 214 girls and 255 boys) were analyzed. Table 3 shows the characteristics of the participants in this study. The study was approved by the institutional review board of the Hokkaido University of Education and the principals of participating schools.

**Table 3:** Characteristics of study participants.

Variables	7th grade	9th grade
*N*	469	
Girls/boys	214/255	
Household income^a^	3 (1)	
Maternal education^a^	3 (1)	
BMI (kg/m^2^)	19 (3)	20 (3)
Lowest GP	3 (1)	3 (1)
Highest GP	4 (1)	4 (1)
Cardiorespiratory fitness	5 (2)	6 (2)
Learning time^a, b^	5 (1)	5 (2)

Values are presented as N or mean (SD).

*BMI* body mass index, *GP* grade point.

^a^These measures were assessed by using a five-point scale: household income, 1 = <2 million yen, 2 = 2 to 4 million yen, 3 = 4 to 6 million yen, 4 = 6 to 8 million yen, 5 = >8 million yen; maternal education, 1 = junior high school, 2 = high school, 3 = vocational school, 4 = junior college, 5 = undergraduate studies; learning time, 1 = <30 min, 2 = 30 min to 1 h, 3 = 1 to 2 h, 4 = 2 to 3 h, 5 = >3 h.

^b^This score is presented as the sum of scores on weekdays and weekends.

### Procedure and measures

The academic year runs from April to March in Japan. We collected data on the participants’ academic performance at the end of the seventh and the ninth grades (i.e., two years apart). Participants’ fitness and out-of-school learning time were assessed in Octobers when they were in the seventh and the ninth grade. The participants’ SES was assessed in October when they were in the seventh grade.

#### Academic performance

The GPs of five academic subjects (Japanese, mathematics, social studies, science, and English) in the national standard curriculum was obtained from schools and used to measure academic performance. Students earn 1–5 points for each subject, with high GPs indicating high academic performance. We analyzed an individual’s lowest and highest GPs at the end of the seventh and ninth grades.

#### Cardiorespiratory fitness

Participants performed a cardiorespiratory fitness test during a physical education class conducted in each school. This fitness test has been adopted by the Ministry of Education, Culture, Sports, Science, and Technology of Japan and annually conducted nationwide. In the test, each school can select either a 20-m shuttle run or an endurance run of 1500 m for boys and 1000 m for girls as a cardiorespiratory fitness test. The data obtained from the cardiorespiratory fitness test was converted into a score ranging from 1 to 10, based on age- and sex-specific normative data for Japanese people^36^, such that high scores indicated high cardiorespiratory fitness.

#### Learning time

Out-of-school learning time was assessed by using a five-point, self-reported questionnaire inquiring about the participants’ out-of-school learning time on weekdays and weekends (from “<30 min/day” to “>3 h/day”). The scores for weekdays and weekends were summed and analyzed. We could obtain learning time data of only 451 participants at baseline and 466 participants at follow-up because of missing values.

#### Body mass index

The data on body weight and height were obtained from schools. We obtained body mass index (BMI) data, calculated as body weight (kg)/height (m^2^), for 468 and 356 participants at baseline and follow-up, respectively, because the follow-up data of one school were missing.

#### Socioeconomic status

SES was assessed only at baseline by using a five-point questionnaire administered to participants’ parents or guardians, which requested the participants’ parents or guardians about their household income (from “<2 million yen” to “>8 million yen”), and maternal educational attainment (ranging from “complete junior high school” to “earned a bachelor’s degree”). We obtained the SES data consisting of household income and maternal educational attainment only from 354 and 361 participants, respectively, due to non-respondents.

### Statistical analysis

Pearson’s correlation analyses were conducted to determine the association between changes in cardiorespiratory fitness and out-of-school learning time with the lowest and the highest GPs. Then, multiple regression analyses with full-information maximum likelihood estimation were conducted for predicting the lowest and highest GPs after controlling for household income, maternal education, sex, change in BMI, and change in learning time. All statistical analyses were conducted with *α* = 0.05 using the R Studio software, version 1.1.463. As mentioned, this study was a reanalysis of our previous study^11^. Therefore, post-hoc power analyses were conducted to determine statistical power using G*Power 3.1.2^37^. The effect size was defined as a small, medium, and large when Cohen’s *f*^2^ = 0.02, 0.15, and 0.35, respectively^38^.

### Reporting summary

Further information on research design is available in the Nature Research Reporting Summary linked to this article.

## Supplementary information

REPORTING SUMMARY

## Data Availability

The datasets generated during and/or analyzed during the current study are available from the corresponding author on reasonable request.

## Code availability

No custom and original software were used in this study.
