# Author Correction: Differential effects of changes in cardiorespiratory fitness on worst- and best- school subjects

**DOI:** 10.1038/s41539-021-00115-6

**Published:** 2021-12-07

**Authors:** Toru Ishihara, Noriteru Morita, Toshihiro Nakajima, Koji Yamatsu, Koichi Okita, Masato Sagawa, Keita Kamijo

**Affiliations:** 1 Graduate School of Human Development and Environment, Kobe University, Kobe, Hyogo Japan; 2 Department of Sports Cultural Studies, Hokkaido University of Education, Iwamizawa, Hokkaido Japan; 3 Department of Teachers Training, Hokkaido University of Education, Sapporo, Hokkaido Japan; 4 Faculty of Education, Saga University, Saga, Saga Japan; 5 Department of Sport Education, Hokusho University, Ebetsu, Hokkaido Japan; 6 University of Tsukuba, Tsukuba, Ibaraki Japan

**Keywords:** Human behaviour, Education, Human behaviour, Education

Correction to: *npj Science of Learning* 10.1038/s41539-021-00086-8, published online 1 April 2021

In this article there was an error in the data reported for “Change in learning time” in Table 2. This was reported as: Change in lowest GP: β = −0.16; SE = 0.05; 95% CI = −0.25 to −0.07; *p* = <0.001; Change in highest GP: β = −0.22; SE = 0.05; 95% CI = −0.31 to −0.13; *p* = <0.001. This has been corrected to: Change in lowest GP: β = 0.16; SE = 0.05; 95% CI = 0.07 to 0.25; *p* = <0.001; Change in highest GP: β = 0.22; SE = 0.05; 95% CI = 0.13 to 0.31; *p* = <0.001. The original article has been corrected.

