# Supplementary material for: Differential effects of changes in cardiorespiratory fitness on worst- and best- school subjects
Source: NPJ Sci Learn. 2021 Apr 1;6:8. doi: 10.1038/s41539-021-00086-8 (PMC8016962; doi:10.1038/s41539-021-00086-8)
Supplement: Supplementary file 1 — REPORTING SUMMARY [file 41539_2021_86_MOESM1_ESM.pdf]

nature research

Corresponding author(s): Noriteru Morita

Last updated by author(s): Nov 30, 2020

# Reporting Summary

Nature Research wishes to improve the reproducibility of the work that we publish. This form provides structure for consistency and transparency in reporting. For further information on Nature Research policies, see our Editorial Policies and the Editorial Policy Checklist.

## Statistics

For all statistical analyses, confirm that the following items are present in the figure legend, table legend, main text, or Methods section.

| n/a | Confirmed | |
|---|---|---|
| ☐ | ☒ | The exact sample size ($n$) for each experimental group/condition, given as a discrete number and unit of measurement |
| ☐ | ☒ | A statement on whether measurements were taken from distinct samples or whether the same sample was measured repeatedly |
| ☐ | ☒ | The statistical test(s) used AND whether they are one- or two-sided<br>*Only common tests should be described solely by name; describe more complex techniques in the Methods section.* |
| ☐ | ☒ | A description of all covariates tested |
| ☐ | ☒ | A description of any assumptions or corrections, such as tests of normality and adjustment for multiple comparisons |
| ☐ | ☒ | A full description of the statistical parameters including central tendency (e.g. means) or other basic estimates (e.g. regression coefficient) AND variation (e.g. standard deviation) or associated estimates of uncertainty (e.g. confidence intervals) |
| ☐ | ☒ | For null hypothesis testing, the test statistic (e.g. $F$, $t$, $r$) with confidence intervals, effect sizes, degrees of freedom and $P$ value noted<br>*Give P values as exact values whenever suitable.* |
| ☒ | ☐ | For Bayesian analysis, information on the choice of priors and Markov chain Monte Carlo settings |
| ☒ | ☐ | For hierarchical and complex designs, identification of the appropriate level for tests and full reporting of outcomes |
| ☐ | ☒ | Estimates of effect sizes (e.g. Cohen's $d$, Pearson's $r$), indicating how they were calculated |

*Our web collection on statistics for biologists contains articles on many of the points above.*

## Software and code

Policy information about availability of computer code

| | |
|---|---|
| Data collection | No software was used for data collection. |
| Data analysis | No custom and original software was used in this study. |

For manuscripts utilizing custom algorithms or software that are central to the research but not yet described in published literature, software must be made available to editors and reviewers. We strongly encourage code deposition in a community repository (e.g. GitHub). See the Nature Research guidelines for submitting code & software for further information.

## Data

Policy information about availability of data

All manuscripts must include a data availability statement. This statement should provide the following information, where applicable:

- Accession codes, unique identifiers, or web links for publicly available datasets
- A list of figures that have associated raw data
- A description of any restrictions on data availability

The datasets generated during and/or analysed during the current study are available from the corresponding author on reasonable request.

# Field-specific reporting

Please select the one below that is the best fit for your research. If you are not sure, read the appropriate sections before making your selection.

☐ Life sciences ☒ Behavioural & social sciences ☐ Ecological, evolutionary & environmental sciences

For a reference copy of the document with all sections, see nature.com/documents/nr-reporting-summary-flat.pdf

# Behavioural & social sciences study design

All studies must disclose on these points even when the disclosure is negative.

| | |
|---|---|
| Study description | This study was performed using a 2-year longitudinal observational study design and analyzed quantitative and qualitative data. |
| Research sample | A total of 469 (boys/girls = 255/214) students aged 12-13 years, who lived in Hokkaido, Japan, participated in this study. These students were from three public schools in different cities in Hokkaido, Japan. |
| Sampling strategy | Recruitment of participants was performed as follows: (1) 20 public junior high schools with 3–6 classes per grade were asked to participate in this study, (2) 14 schools declined participation, and (3) two schools were lost to the 2-year follow-up. Finally, 469 children completed all measurements and questionnaires. Data collected from these students were then analyzed.<br>Power analyses with post-hoc procedure were conducted to determine statistical power using G*Power 3.1.2. The effect size was defined as small, medium, and large when Cohen's f2 = .02, .15, and .35, respectively. |
| Data collection | Physical fitness and body size measures of children were recorded by teachers using pen and paper and then inputted to a personal computer. The grade point of each academic subject, as a measure of academic performance, was evaluated by teachers of each academic subject throughout a school year, based on class activities, and reflection papers, and paper test scores. Questionnaires for daily lifestyle and family's socioeconomic status were answered by children themselves and their parents/guardians, respectively (see the Methods section). |
| Timing | Baseline data for body size, physical fitness, and lifestyle were assessed in May, June, and October 2012, respectively. Grade points of academic subjects in the 7th grade were determined at the end of the school year, i.e., March 2013 (in Japan, the school year starts in April and ends in March of the following year). Follow-up data for body size, physical fitness, lifestyle, and grade points were collected in the same months after 2 years (May 2014 to March 2015). |
| Data exclusions | No data were excluded from this study except for children who lost data for grade points and physical fitness. |
| Non-participation | Two schools were dropped out at follow-up measurement because of missing data. |
| Randomization | Participants were not allocated into experimental groups. |

# Reporting for specific materials, systems and methods

We require information from authors about some types of materials, experimental systems and methods used in many studies. Here, indicate whether each material, system or method listed is relevant to your study. If you are not sure if a list item applies to your research, read the appropriate section before selecting a response.

## Materials & experimental systems

| n/a | Involved in the study | |
|---|---|---|
| ☒ | ☐ | Antibodies |
| ☒ | ☐ | Eukaryotic cell lines |
| ☒ | ☐ | Palaeontology and archaeology |
| ☒ | ☐ | Animals and other organisms |
| ☐ | ☒ | Human research participants |
| ☒ | ☐ | Clinical data |
| ☒ | ☐ | Dual use research of concern |

## Methods

| n/a | Involved in the study | |
|---|---|---|
| ☒ | ☐ | ChIP-seq |
| ☒ | ☐ | Flow cytometry |
| ☒ | ☐ | MRI-based neuroimaging |

## Human research participants

Policy information about studies involving human research participants

| | |
|---|---|
| Population characteristics | See above. |
| Recruitment | See above. |

| | |
|---|---|
| Ethics oversight | The ethical review board of the Hokkaido University of Education. |

Note that full information on the approval of the study protocol must also be provided in the manuscript.
